# Peripheral Neutrophil Activation and Extracellular Trap Formation in Amyotrophic Lateral Sclerosis

**DOI:** 10.1002/acn3.70431

**Published:** 2026-05-14

**Authors:** Lillia A. Baird, Haley McQuown, Jihyun Park, Samuel J. Teener, Ian F. Webber‐Davis, Andrew D. Carter, Dae‐Gyu Jang, Eva L. Feldman, Stephen A. Goutman, Benjamin J. Murdock

**Affiliations:** ^1^ Department of Neurology University of Michigan Ann Arbor Michigan USA; ^2^ Graduate Program in Immunology University of Michigan Ann Arbor Michigan USA

**Keywords:** ALS, immune system, motor neuron disease, neutrophils

## Abstract

**Objectives:**

Peripheral neutrophil levels in amyotrophic lateral sclerosis (ALS) inversely correlate with survival, suggesting a role for neutrophils in disease progression. Here, we characterize markers of several neutrophil activation pathways and evaluate their associations with survival to identify potential mechanisms of disease.

**Methods:**

Blood samples were obtained from participants at the University of Michigan ALS Clinic or from healthy controls. Ex vivo neutrophil extracellular trap (NET) formation was quantified via image analysis of primary neutrophils. Neutrophil function markers of general activation (calprotectin), migration (matrix‐metalloproteinase 9 [MMP9]), and degranulation (neutrophil gelatinase‐associated lipocalin [NGAL]) were then quantified in plasma via ELISA; NET formation (double‐stranded DNA [dsDNA]) was assessed via fluorescence assay. These markers were then associated with ALS survival using Cox proportional hazard regression models, and analyses were stratified by sex.

**Results:**

Spontaneous ex vivo NET formation (*N* = 20 controls, 66 ALS) was increased in ALS (1.0% vs. 9.7%; *p* = 0.017). In plasma (*N* = 233 controls, 178 ALS), calprotectin (294 vs. 372 ng/mL; *p* < 0.001), MMP9 (106 vs. 152 ng/mL; *p* < 0.001), and NGAL (61 vs. 66 ng/mL; *p* = 0.01) were elevated in ALS. Calprotectin, MMP9, and NGAL levels were not associated with ALS survival; however, dsDNA was associated with poorer ALS survival but only in females (HR = 1.77 [95% CI, 1.20–2.61]; *p* = 0.004).

**Interpretation:**

Neutrophil function is altered in ALS, and NET formation is a potential mechanism by which neutrophils contribute to ALS, particularly in females.

## Graphical abstract

Markers of neutrophil activation are increased in plasma during ALS, and markers of NET formation associate with ALS survival.

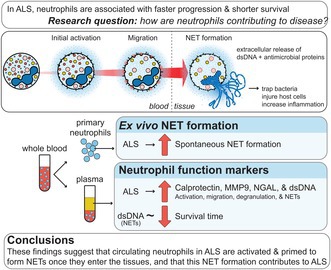

## 1 Introduction

Amyotrophic lateral sclerosis (ALS) is an incurable and rapidly fatal motor neuron disease causing progressive cranial, limb, and respiratory weakness [1]. Among the complex pathophysiology of ALS [1, 2], immune system dysregulation is consistently implicated [3, 4], and the immune system appears to contribute to ALS pathology both in mouse models [5, 6] and human patients [3, 7]. Multiple immune cells are altered in preclinical and clinical ALS studies including T cells, natural killer (NK) cells, and neutrophils [3, 4, 5, 8, 9]. These immune cells infiltrate the central nervous system tissue [5, 6] and are dysregulated in the peripheral blood [3, 9, 10, 11]. Moreover, immunomodulatory drugs are increasingly available, though their use in ALS has not yet proven successful [12]. Further studies examining the underlying immune mechanisms in ALS may lead to new mechanism‐based strategies with a greater chance of therapeutic efficacy.

**FIGURE 1:**
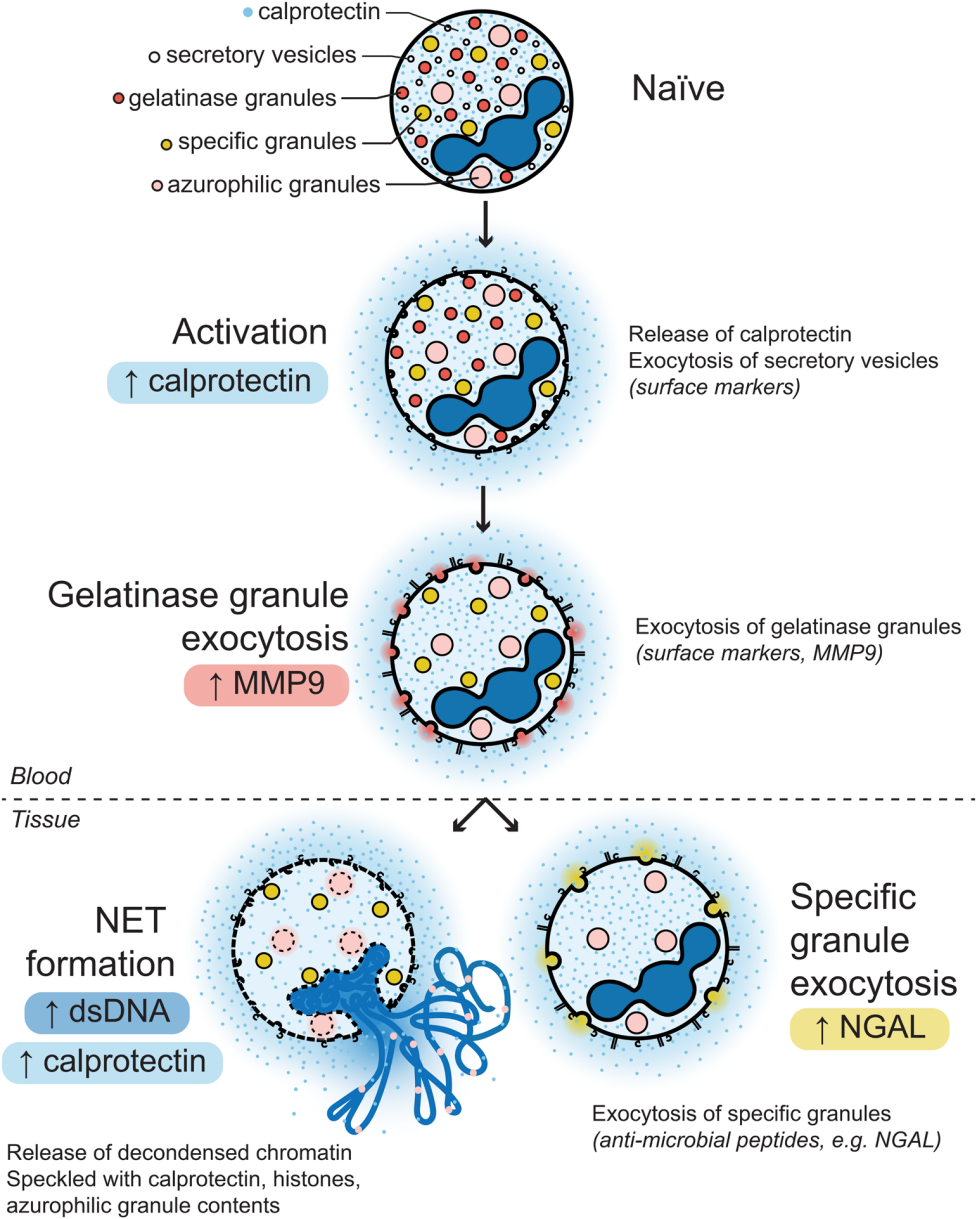
Overview of progressive neutrophil activation and associated markers. Naïve neutrophils: Most protein synthesis occurs in the bone marrow, allowing for the release of fully armed neutrophils. Calprotectin (S100A8/S100A9), found in the cytoplasm, comprises half the protein content of the neutrophil. Additional proteins are stored in the granules: Secretory, gelatinase, specific, and azurophilic. Activation: A low level of signaling, including brief contact with activated endothelial cells or sensing of danger‐associated molecular patterns (DAMPs), is sufficient for calprotectin release and secretory vesicle endocytosis. Surface proteins, including integrins and signaling receptors, comprise the majority of secretory vesicle contents, with relatively few secreted proteins. These allow for firm adhesion of the neutrophil to the endothelium, continuing the migration cascade. Autocrine and paracrine signaling by released calprotectin activates cells, including neutrophils and endothelial cells. Gelatinase granule exocytosis: Further signaling, including that from continued interactions with the endothelium, stimulates the release of gelatinase granules. These granules contain similar surface markers to secretory vesicles, with the addition of matrix metalloproteinases, primarily MMP9; these can also decrease blood–brain barrier (BBB) and blood spinal‐cord barrier (BSCB) integrity [18]. NET formation: Strong, sustained signaling can result in NET formation through a variety of pathways. In classical NET formation, azurophilic granule proteins, including neutrophil elastase, migrate to the nucleus, leading to chromatin decondensation. This decondensed chromatin, speckled with histones, azurophilic granule contents, and calprotectin, is then released extracellularly and can be detected as circulating dsDNA. Specific granule exocytosis: Strong signaling can also result in complete activation, including the degranulation of specific granules, reactive oxygen species (ROS) generation, and mobilization of the azurophilic granules to fuse with phagosomes. Specific granules contain potent antimicrobial proteins, including lysozyme, lactoferrin, hCAP18, and NGAL, capable of harming host cells in addition to pathogens [19, 20, 21, 22]. Abbreviations: S100A8—S100 calcium‐binding protein A8; S100A9—S100 calcium‐binding protein A9; DAMP—danger‐associated molecular pattern; MMP9—matrix metalloproteinase 9; NET—neutrophil extracellular trap; dsDNA—double‐stranded DNA; ROS—reactive oxygen species; hCAP18—human cationic antimicrobial protein; NGAL—neutrophil gelatinase‐associated lipocalin 2.

Our previous studies [3, 4, 8], as well as those from other groups [13, 14, 15, 16, 17], highlight important associations between neutrophils and ALS progression, particularly in female patients [8, 17]. Neutrophils are highly prevalent innate immune cells, with functions including activation, migration, degranulation, and neutrophil extracellular trap (NET) formation (Figure 1) [19, 20, 21]. While these are essential for pathogen control and inflammation resolution, dysregulation of these functions is associated with a number of diseases, including chronic obstructive pulmonary disease, rheumatoid arthritis, and systemic lupus erythematosus [19]. Moreover, neutrophils are also implicated in the pathogenesis of neurodegenerative diseases such as Alzheimer's disease [18, 23] and multiple sclerosis [24], suggesting a common role in neurodegeneration. Despite these observations, these cells are understudied in ALS, likely due to difficulties with overall cellular stability and in vitro manipulation [25, 26]. Therefore, the mechanisms underlying the association between neutrophils and ALS require further exploration.

Given the likely role neutrophils play in ALS progression [8, 13, 14, 15, 16], we sought to better characterize the specific neutrophil functions that may contribute to ALS pathogenesis through two complementary approaches. Ex vivo, we compared spontaneous and maximal NET formation of primary neutrophils from controls and participants with ALS. Next, neutrophil function markers were quantified in plasma; these included markers indicative of activation (calprotectin [22]), migration (matrix metalloproteinase‐9 [MMP9] [19]), degranulation (neutrophil gelatinase‐associated lipocalin [NGAL] [19]), and NETs (double‐stranded DNA [dsDNA] [19, 27]). Levels were compared between controls and participants with ALS, assessed for associations with ALS survival, and analyses were stratified by sex.

## 2 Methods

### 2.1 Study Participants

This study comprised samples from two participant cohorts: Samples of freshly collected blood used for evaluating ex vivo NET formation and samples of existing frozen plasma for evaluating neutrophil function markers. Study protocols are published [4, 7, 8]. Briefly, all participants with an El Escorial diagnosis of ALS seen in the ALS Clinic at the Scott Pranger ALS Center at the University of Michigan were invited to participate. Controls were recruited via electronic study announcements. Controls were excluded if they had a personal or family history of ALS or another neurodegenerative disease, chronic inflammatory disease, current infection, or use of immunomodulatory medication. Age and sex were recorded for all participants. For participants with ALS, disease characteristics were extracted from medical records, including age at onset and diagnosis, onset segment, and El Escorial criteria. ALS Functional Rating Scale Revised (ALSFRS‐R), administered by trained evaluators blinded to study participation, was also abstracted from the medical records at routine longitudinal clinic visits.

### 2.2 NET Quantification Using DNA Area and NETosis Analysis (DANA)

Neutrophils were isolated from blood collected in sodium‐heparin‐coated tubes (BD Biosciences) via negative selection using an EasySep direct human neutrophil isolation kit per the manufacturer's instructions (StemCell Technologies, Vancouver, Canada). Fresh samples collected from study participants between May 2023 and March 2024 were used for this analysis, regardless of the time between diagnosis and sample collection. The effects of sample processing were minimized with well‐established protocols followed consistently; all samples were kept on ice until processing, processing occurred within 2 h of sample collection, and the brand and type of consumables and reagents remained consistent throughout. The same four researchers performed all sample processing across the duration of the study and were blinded to case status.

Following isolation from peripheral blood, primary neutrophils were resuspended at 10^5^ cells/mL in assay buffer (1× HBSS++, 10 nM HEPES, 0.05% heat‐inactivated FBS) and stimulated with 100 nM phorbol 12‐myristate 13‐acetate (PMA) in DMSO or DMSO alone as a vehicle control to measure spontaneous NET formation. Cells were plated in duplicate on CultureWell coverslips (Invitrogen) previously treated with poly‐L‐lysine (Sigma‐Aldrich, St. Louis, USA) for 15 min and incubated for 2 or 4 h at 37°C with 5% CO_2_. Slides were fixed with methanol for 10 min at −20°C, rinsed with HBSS, stained with 500 nM Sytox green (Invitrogen) for 10 min, rinsed, and mounted to slides using ProLong Gold (Invitrogen) to visualize DNA. Slides were then imaged on a Ti2 widefield microscope (Nikon Instruments, Melville, USA) with excitation at 488 nm and a 20× objective. Imaging was semi‐automated to reduce bias, with 8 images taken at pre‐defined locations around the approximate center of the well. Laser power and exposure time were kept consistent across all slides. Slides for representative images were stained with wheat germ agglutinin (Invitrogen) in addition to Sytox and were imaged at 40×. Image analysis was performed using DNA Area and NETosis Analysis (DANA) [28]; the rolling ball method was used for background subtraction. Briefly, regions of interest (ROIs) were identified and exported; statistics were grouped by participant. ROIs with a raw integrated density below 400,000 (fragments) and those exceeding 1.8 standard deviations above the mean integrated density (multiples) were excluded from analysis. ROIs were classified as NETs if the relative area was greater than 9 times the average area of the five smallest cells [28].

### 2.3 Plasma Neutrophil Function Marker Quantification

Previously frozen plasma samples obtained from study participants at study entry were used in the analysis. Briefly, blood in EDTA‐coated tubes (BD Biosciences, San Jose, USA) was centrifuged at 2,000 g for 10 min at 4°C before being aliquoted and stored at −80°C in the University of Michigan ALS Biorepository until time of analysis. All plasma samples were obtained between January 2021 and July 2024.

Calprotectin, MMP9, and NGAL were quantified via ELISA (R&D Systems, Minneapolis, USA) following the manufacturer's protocols. Plasma was diluted 1:1000 in sample diluent for calprotectin, 1:250 for MMP9, and 1:100 for NGAL to ensure values within the limits of detection. All samples were assayed using capture antibody, detection antibody, and standard reagents from the same lot. Reagent diluent (R&D Systems), sample diluent (reagent diluent, 1% heat‐inactivated FBS), and wash buffer (1× PBS, 0.05% tween‐20) were prepared fresh daily. Absorbance was measured at 450 and 570 nm using a Synergy HTX plate reader (BioTek Instruments, Winooski, USA) running Gen 5 software (version 3.03). Wavelength correction was performed by subtracting the blanked 570 nm value from the 450 nm blanked value. A four‐parameter logistic standard curve was calculated and used to find sample concentrations.

dsDNA was quantified using the Quant‐iT high‐sensitivity dsDNA kit (Invitrogen, Waltham, USA, Q33120) in undiluted plasma following the product protocol. Fluorescence was measured with excitation at 485/20 nm and emission at 528/20 nm using a Synergy HTX plate reader (BioTek Instruments) running Gen 5 software (version 3.03). A linear standard curve was calculated and used to identify sample concentrations.

For both ELISA and dsDNA analyses, an internal control was used to ensure assay rigor and consistency across plates. To do so, de‐identified peripheral blood was obtained from healthy controls through the Platelet Pharmacology and Physiology Core (HUM107120) at the University of Michigan. No protected health information was provided, only participant age and sex. Blood was collected via peripheral venipuncture and stored at room temperature until the time of processing (< 1 h). Blood was layered over Ficoll‐Paque (Cytiva, Marlborough, USA) and centrifuged at 400 g for 20 min with acceleration set to 3 and brake set to 2. The top layer of plasma was transferred to a separate tube and stored at −80°C. Samples from three separate individuals were thawed, pooled, and thoroughly vortexed. Samples were then aliquoted and stored at −80°C until the time of ELISA and dsDNA analyses.

For both ELISA and dsDNA analyses, the Optimal Sample Assignment Tool (OSAT) was used to determine assignments and layout on the 96‐well plate; samples were distributed by disease status, sex, and age to reduce batch effects [29]. All samples and standards were run in duplicate. An internal control and blanks were included on every plate. Background noise was removed by subtracting the average of the blank wells from each well on the plate prior to wavelength correction and standard curve calculations.

### 2.4 Flow Cytometry

Neutrophil markers and immune population counts were obtained via flow cytometry as previously described [4].

### 2.5 Statistical Analysis

Summary statistics: Demographics and ALS disease features were summarized with median and interquartile range (IQR) for continuous variables and counts and percentages for categorical variables. Statistical significance between groups was determined via the Wilcoxon rank sum test for continuous variables between two groups, and the chi‐square test for categorical variables. Mean and coefficient of variation (CV) were calculated from replicates (NET quantification) and duplicates (neutrophil function markers). Samples with a CV greater than 20% were excluded (Figure S1) [30]. For neutrophil function markers, mean‐centering using the internal controls was used to normalize across plates [31]. Concentrations above or below the limits of detection (LOD) were set to the LOD + 1 or LOD 1, respectively. Means were multiplied by the dilution factor and converted to ng/mL. Outliers were defined as greater than the sum of the 3rd quartile and 3 interquartile ranges (Q3 + 3xIQR) [32] and were imputed as this maximum value. For NET quantification, cell count, and onset segment sensitivity analyses, statistical significance was tested with the Wilcoxon rank sum test for continuous variables between two groups and the Kruskal–Wallis test for continuous variables with three or more groups. Correlations were calculated using Spearman's rank correlation coefficient. Age matching was performed using a nearest neighbor algorithm, allowing a 3‐year age gap between matched control and ALS pairs. Associations between neutrophil function markers and ALS case status were tested using logistic regression models adjusted by age and sex, with a single neutrophil function marker as a predictor and ALS case status as the outcome variable.

Survival models: Cox proportional hazard regression was performed for time from sample to death, adjusted for important clinical covariates including sex, age at diagnosis, El Escorial criteria, ALSFRS‐R at sample, onset segment, diagnostic delay, and the time difference between diagnosis and sample dates. Analyses were then stratified by sex. Statistical analyses were performed using R (version 5.1.4).

### 2.6 Standard Protocol Approvals, Registrations, and Patient Consents

This study used samples and data collected under HUM28826 and HUM107120, approved by the Institutional Review Boards of the University of Michigan Medical School. All participants provided written informed consent.

## 3 Results

### 3.1 Ex Vivo Quantification of NET Formation

**FIGURE 2:**
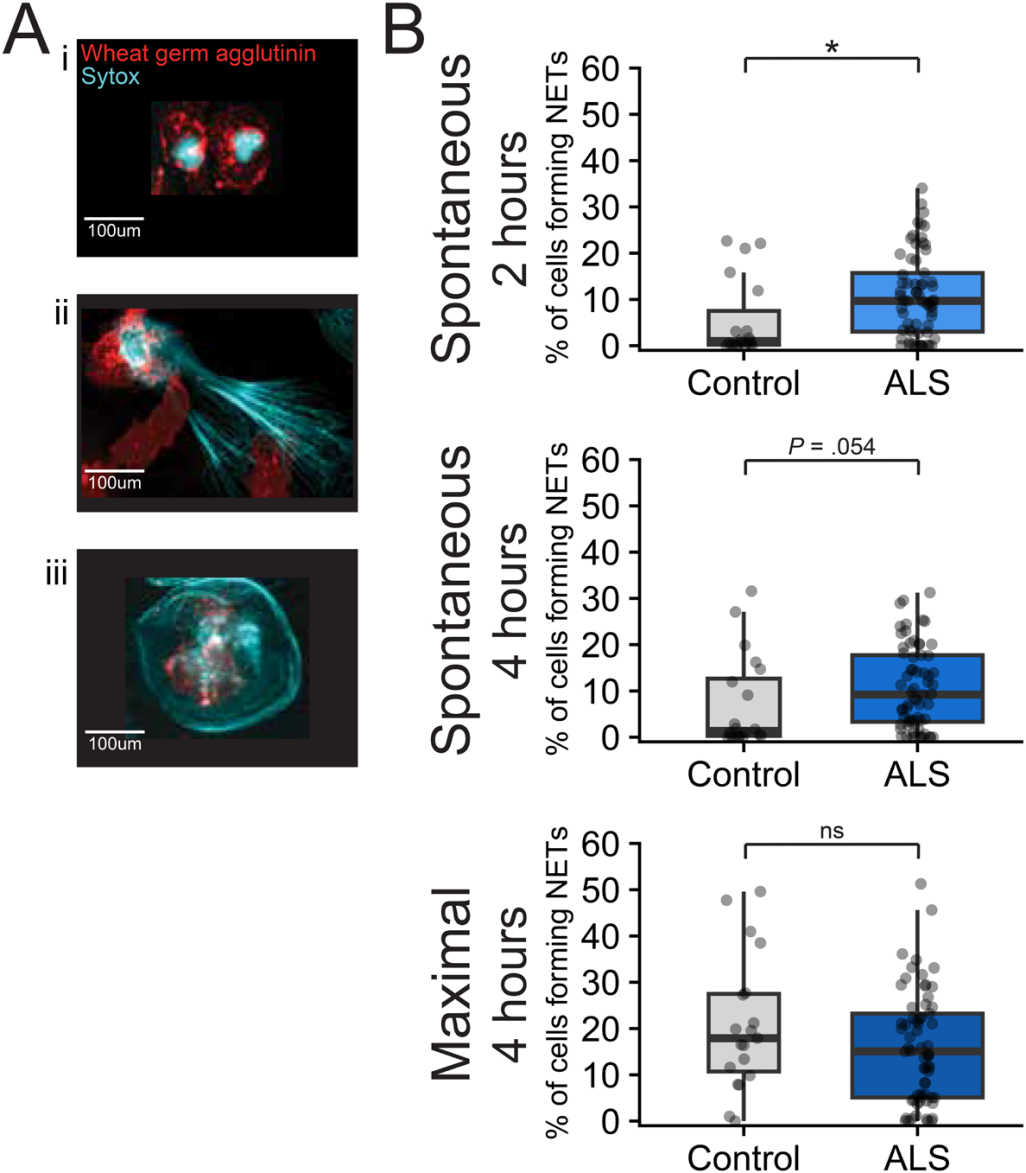
Quantification of neutrophil extracellular trap (NET) formation by primary neutrophils from controls and participants with ALS. Primary neutrophils were isolated via negative selection from the peripheral blood of participants in the NET cohort. Spontaneous NET formation was assessed in cells treated with vehicle DMSO for 2 or 4 h. Maximal NET formation was assessed in cells with 100 nM PMA for 4 h. An average of 16 images from pre‐determined locations on the slide were analyzed per condition using DNA Area and NETosis Analysis (DANA). (A) Representative images of neutrophils stained with Sytox (DNA) and wheat germ agglutinin (sialic acid, cell membrane, and intracellular marker). (i) Neutrophils not forming NETs were characterized by compact, multi‐lobular nuclei. (ii, iii) Neutrophils forming NETs were characterized by an increase in DNA area resulting from chromatin decondensation and extracellular release. (B) Proportion of neutrophils forming NETs between controls and participants with ALS, stratified by timepoint and condition. *p*‐values from the Wilcox test, * ≤ 0.05. Abbreviations: NET—neutrophil extracellular trap; DMSO—dimethyl sulfoxide; PMA—phorbol 12‐myristate 13‐acetate; DANA—DNA area and NETosis analysis.

As NET formation is implicated in neurodegenerative disease pathogenesis [18, 23, 24, 33], we began by directly examining the capacity of ALS neutrophils to form NETs in 86 participants: 20 controls and 66 participants with ALS (Table 1). Control participants were younger (*p* = 0.029), and although not statistically significant, had a higher frequency of females compared to ALS. For participants with ALS, samples were collected a median of 8.3 m after diagnosis. Neutrophils were assessed at 2 and 4 h for spontaneous NET formation, and at 4 h with stimulation to assess maximal NET formation capacity. Neutrophils were categorized as either not forming NETs (Figure 2Ai, characterized by a compact nucleus) or as forming NETs with decondensed chromatin; chromatin in NET‐forming cells could either be ejected through openings in the plasma membrane (Figure 2Aii) or released during cell lysis (Figure 2Aiii).

**TABLE 1:** Demographics and clinical characteristics of participants in the NET cohort.

	Control (*n* = 20)	ALS (*n* = 66)	*P*
**Sex**			0.060^a^
Male	9 (45%)	45 (68%)	
Female	11 (55%)	21 (32%)	
**Age at sample (years)**	61.0 (57.3–66.5)	67.0 (58.9–73.2)	0.029^b^
**Race**			
White	20 (100%)	62 (94%)	
Black or African American	0 (0%)	3 (4.5%)	
Asian	0 (0%)	1 (1.5%)	
**Age at onset (years)**		64.0 (55.5–70.4)	
**ALSFRS‐R at sample**		34.0 (27.5–39.5)	
**Symptom onset to diagnosis (months)**		11.9 (6.4–24.6)	
**Diagnosis to sample (months)**		8.3 (6.5–22.2)	
**El Escorial Criteria**			
Definite		23 (35%)	
Probable		27 (42%)	
Probable, lab supported		13 (20%)	
Possible		1 (1.5%)	
Suspected		1 (1.5%)	
**Onset segment**			
Lumbar		24 (37%)	
Cervical		28 (43%)	
Bulbar		10 (15%)	
Thoracic		1 (1.5%)	
Respiratory		2 (3.5%)	

*Note:* N missing: Age at sample—2 (ALS); age at onset—1; ALSFRS‐R at sample—2; Symptom onset to diagnosis—1. *N* (%); Median (IQR).

Abbreviations: ALSFRS‐R, amyotrophic lateral sclerosis functional rating score—revised; IQR, inter‐quartile range.

^a^ Pearson's Chi‐squared test.

^b^ Wilcoxon rank sum test.

Relative to controls, neutrophils from ALS participants formed more spontaneous NETs at 2 h (controls: Median 1.0% [IQR 0.2–11.9]; ALS: 9.7% [IQR 3.0–15.7]; *p* = 00.017), with similar results at 4 h (controls: 1.4% [IQR 0.3–13.3]; ALS: 9.2% [IQR 3.3–17.7]; *p* = 0.054) (Figure 2B; Table S1). There was no difference in maximal NET formation (controls: 18.7% [IQR 10.7–33.1]; ALS: 15.1% [IQR 5.1–23.2]; *p* = 0.2). As age can impact neutrophil function, we also determined whether age was associated with spontaneous or maximal NET formation; no major effects of age on NET formation were observed (Figure S2A). We also examined whether NET formation was associated with the onset segment; NET formation was similar regardless of onset site (Figure S2B). Together, these data show that neutrophils from ALS participants are more prone to spontaneous NET formation, though the maximum capacity of neutrophils to form NETs is not different between ALS and control participants.

### 3.2 Neutrophil Function Marker Analysis

Alongside the ex vivo analysis, we also quantified plasma calprotectin, MMP9, NGAL, and dsDNA levels in biobanked samples. This cohort included samples from 233 controls and 178 participants with ALS (Table 2). Groups did not differ by sex, although controls were slightly younger than participants with ALS. Among ALS participants, disease characteristics reflected a typical ALS cohort. Samples were collected shortly following diagnosis to focus on neutrophil function early in clinical disease. Time from diagnosis to the time of sample collection was only 2.3 months, and the median ALSFRS‐R at the time of sample collection was 36. This is in comparison to the NET cohort, which had a median of 8.3 months from diagnosis to the time of sample collection and a median ALSFRS‐R of 34 at the time of sample collection (Table S2). When this group was stratified by sex, clinical features were overall similar except for ALSFRS‐R at the time of sample collection, which was slightly lower in females (males: 37 [IQR 32–41]; females: 34 [IQR 27–40]; *p* = 0.035) (Table S3). Prior to analysis, neutrophil function marker data were assessed for quality and were normalized to correct for batch effects (Figure S1).

**TABLE 2:** Demographics and clinical characteristics of participants in the neutrophil function marker cohort.

	Control (*n* = 233)	ALS (*n* = 178)	*P*
**Sex**			0.7^a^
Male	140 (60%)	109 (62%)	
Female	93 (40%)	69 (38%)	
**Age at sample (years)**	62.2 (56.3–68.6)	66.8 (60.5–72.9)	< 0.001^b^
**Race**			0.011^a^
White	229 (97.4%)	171 (96%)	
Black or African American	0 (0%)	5 (3%)	
Asian	5 (2.1%)	1 (0.5%)	
American Indian and Alaska Native	0 (0%)	1 (0.5%)	
Unknown or Not Reported	1 (0.5%)	0 (0%)	
**Age at onset (years)**		65.2 (58.5–71.1)	
**ALSFRS‐R at sample**		36 (30–41)	
**Symptom onset to diagnosis (months)**		11.5 (6.7–18.0)	
**Diagnosis to sample (months)**		2.3 (0.8–5.1)	
**Survival from sample (months)**		13.3 (6.8–20.4)	
**Follow‐up from sample (months)**		16.9 (8.9–25.7)	
**Death event**		95 (52%)	
**20% progression or death event**		95 (52%)	
**El Escorial Criteria**			
Definite		72 (40%)	
Probable		63 (36%)	
Probable, lab supported		37 (21%)	
Possible		3 (2%)	
Suspected		2 (1%)	
**Onset segment**			
Lumbar		71 (40%)	
Cervical		58 (33%)	
Bulbar		41 (23%)	
Thoracic		4 (2%)	
Respiratory		3 (2%)	

*Note:* N missing: Age at onset—3; ALSFRS‐R at sample—1; Symptom onset to diagnosis—6; Diagnosis to sample—4; El Escorial criteria—1; onset segment—1. *N*(%); Median (IQR).

Abbreviations: ALSFRS‐R, amyotrophic lateral sclerosis functional rating score – revised; IQR, inter‐quartile range.

^a^ Pearson's Chi‐squared test.

^b^ Wilcoxon rank sum test.

Next, correlations between calprotectin, MMP9, NGAL, and dsDNA were examined to ensure that neutrophil marker levels followed the expected patterns and to ensure the validity of the subsequent analyses (Figure S3A). There was moderate correlation between calprotectin and the other markers, and weak correlation between MMP9 and NGAL and between NGAL and dsDNA (Figure S3B), demonstrating that marker levels followed the expected patterns. Additionally, the number of neutrophils in the peripheral blood was correlated with calprotectin (*ρ* = 0.27; *p* < 0.001), MMP9 (*ρ* = 0.36; *p* < 0.001), and NGAL (ρ = 0.25; *p* < 0.001), indicating that neutrophils are likely the primary source of these markers.

**FIGURE 3:**
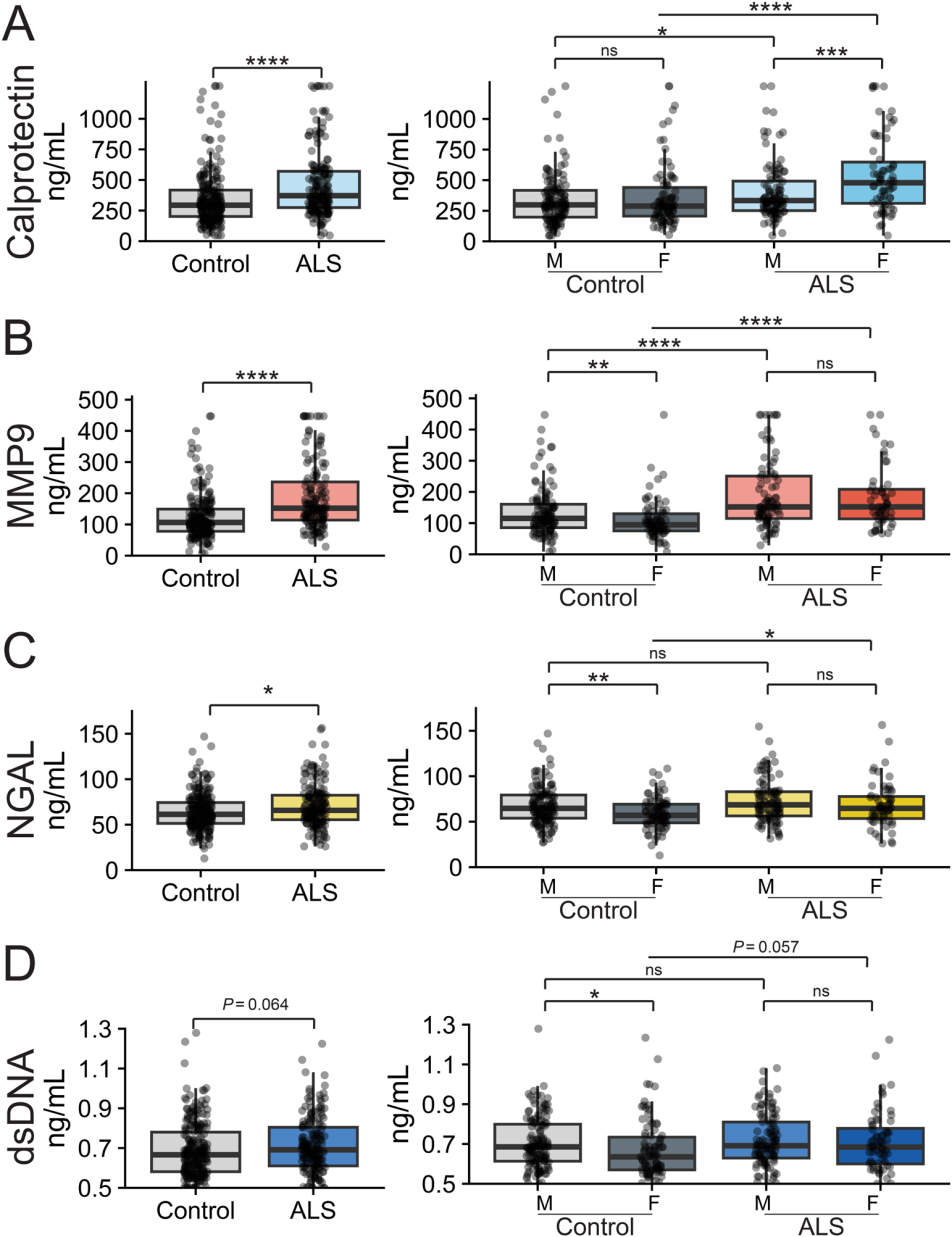
Quantification of neutrophil function markers in plasma in controls and participants with ALS. Plasma concentrations of calprotectin (A), MMP9 (B), NGAL (C), and dsDNA (D) were assessed using ELISA (calprotectin, MMP9, NGAL) and Quant‐iT (dsDNA, Invitrogen), compared between control and ALS participants, and stratified by sex. *p*‐values from Wilcox test and Kruskal–Wallis test, * ≤ 0.05, ** ≤ 0.01, *** ≤ 0.001, **** ≤ 0.0001. Abbreviations: MMP9—matrix metalloproteinase 9; NGAL—neutrophil gelatinase‐associated lipocalin 2; dsDNA—double‐stranded DNA.

Neutrophil function marker levels were then compared between controls and participants with ALS. Compared to controls, participants with ALS had elevated plasma levels of calprotectin (control: 294 ng/mL [IQR 200–418]; ALS: 372 ng/mL [IQR 272–573]; *p* < 0.001), MMP9 (control: 106 ng/mL [IQR 78–149]; ALS: 152 ng/mL [IQR 113–237]; *p* < 0.001), and NGAL (control: 61 ng/mL [IQR 51–74]; ALS: 66 ng/mL [IQR 55–82]; *p* = 0.01) (Figure 3A–C, Table S4). There was also a trend toward increased dsDNA in participants with ALS (control: 0.67 ng/mL [IQR 0.58–0.79]; ALS: 0.69 ng/mL [IQR 0.61–0.80]; *p* = 0.064) (Figure 3D). Data were subsequently stratified by sex: Calprotectin was elevated in female participants with ALS relative to female controls as well as male ALS participants. Female ALS participants showed significantly increased MMP9 and NGAL relative to female controls and a trend toward increased dsDNA; male ALS participants showed increased calprotectin and MMP9 relative to male controls but no increase in NGAL or dsDNA. No significant differences in MMP9, NGAL, or dsDNA were observed between male and female participants with ALS. Next, to verify that differences between controls and participants with ALS were not due to differences in participant age, we performed sensitivity analyses comparing three different logistic regression models: The complete cohort without adjustment for age and sex, the complete cohort with adjustment for age and sex, and a smaller, age‐matched cohort without adjustment for age and sex. No significant differences in demographics were observed between the complete cohort and age‐matched subsample (Table S5), and the odds ratios and statistical significance were similar across all three models, indicating that the observed differences were not due to participant age (Table S6). Together, these data indicate that neutrophil activation, migration, and degranulation are likely increased in ALS, particularly in females.

### 3.3 Association of Neutrophil Function Markers With Disease

**FIGURE 4:**
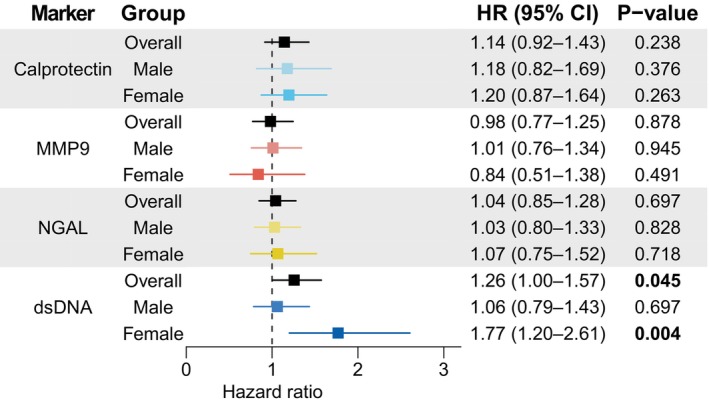
Regression models for survival in all participants with ALS and stratified by sex. Hazard ratios for calprotectin, MMP9, NGAL, and dsDNA were calculated using a Cox regression model for time from sample date to death. Models were adjusted for sex, age at diagnosis, El Escorial criteria, ALSFRS‐R at sample, onset segment, diagnostic delay, and the time difference between diagnosis and sample dates. Analyses were also stratified by sex. Abbreviations: MMP9—matrix‐metalloproteinase 9; NGAL—neutrophil gelatinase‐associated lipocalin‐2; dsDNA = double‐stranded DNA; ALSFRS‐R—amyotrophic lateral sclerosis functional rating score—revised. *p*‐values in bold are < 0.05.

Finally, we associated neutrophil function markers in the plasma with ALS survival. To do so, Cox proportional hazard regression models were generated to determine whether the markers of neutrophil function were associated with reduced ALS survival (Figure 4). To control for clinical heterogeneity, models were adjusted for age at diagnosis, diagnostic delay, time difference between diagnosis and sample dates, El Escorial criteria, ALSFRS‐R at sample, and onset segment. Unexpectedly, we found that calprotectin, MMP9, and NGAL levels in the plasma did not associate with ALS survival within the cohort of ALS participants. However, higher concentrations of plasma dsDNA were associated with poorer survival (HR = 1.26 [95% CI, 1.00–1.57]; *p* = 0.045). We also stratified the data by sex to determine if associations may be sex‐specific. As with the overall analysis, there were no significant associations between calprotectin, MMP9, and NGAL and survival in male or female ALS participants. Strikingly, however, the associations between dsDNA and survival were driven entirely by female participants (survival: HR = 1.77 [95% CI, 1.20–2.61]; *p* = 0.004), with no associations found in male participants. To verify that the onset segment was not driving these observations, data were also stratified by onset segment; stratification of the data did not identify any differences due to onset segment (Figure S4). Together, these data suggest that NET formation is associated with ALS survival, but only in females.

## 4 Discussion

The current study characterized neutrophil phenotypes in ALS, finding that spontaneous NET formation and plasma concentrations of calprotectin, MMP9, and NGAL were elevated in individuals with ALS, particularly females. dsDNA, a marker of NET formation, was also associated with survival in female participants in ALS. These findings are consistent with the current literature suggesting that neutrophils in ALS are more activated [11, 34], associate with poorer disease outcomes [4, 8], and are more prone to NET formation [11, 35, 36]. They also support prior studies linking neutrophils to ALS risk and survival, particularly females [4, 8].

We first demonstrated that spontaneous NET formation is increased ex vivo in neutrophils isolated from peripheral blood in ALS, suggesting that neutrophils from individuals with ALS are primed for NET formation. As illustrated in Figure 1, NET formation requires substantial activation, occurring after initial activation and gelatinase granule exocytosis. However, not all neutrophils will form NETs when stimulated [37]; we found similar maximal NET formation rates in control and ALS samples. This indicates that the increase in spontaneous NET formation in ALS is not due to a change in overall capacity for NET formation, but increased activation of neutrophils in the periphery. Increased NET formation at baseline may therefore be a result of the general increase in inflammation observed in ALS, which includes dysregulated immune cell populations and increased levels of proinflammatory cytokines [38]. Further studies will be required to elucidate the underlying mechanisms.

Next, we utilized frozen plasma samples to assess markers of neutrophil function. This approach was chosen for several reasons. First, we were able to quantify multiple neutrophil markers, allowing us to assess multiple mechanisms that may be contributing to ALS. Second, isolating neutrophils from blood can alter their function; therefore, neutrophil function markers in plasma may more closely reflect neutrophil function in circulation compared to neutrophils subjected to ex vivo manipulation. Third, the use of these markers bypassed logistical difficulties associated with ex vivo studies, allowing a more robust analysis with a larger cohort. Fourth, the large pool of available plasma samples from the University of Michigan ALS Biorepository allowed us to select samples taken soon after participant diagnosis, synchronizing the study and increasing the rigor and reproducibility of the analyses. Finally, the larger cohort allowed us to stratify the analyses by sex, as we have previously shown that the role of neutrophils in ALS may be more pronounced in females [3, 8].

Calprotectin, MMP9, NGAL, and dsDNA were chosen as markers of neutrophil function. These markers cover multiple steps in sequential neutrophil activation (Figure 1), together indicating general activation (calprotectin), migration (calprotectin & MMP9), degranulation (MMP9 & NGAL), and NET formation (dsDNA & calprotectin) (Figure S3A). Though expression of calprotectin, MMP9, and NGAL is not limited to neutrophils, neutrophils are the primary source in the periphery [39, 40], and concentrations were correlated with neutrophil count (Figure S3B). Similarly, any cell could release dsDNA, but neutrophils account for approximately 30%, the highest proportion by cell‐type [40].

We found that calprotectin, MMP9, and NGAL were elevated in the plasma of participants with ALS, while dsDNA was not. These findings suggest increased activation, migration, and degranulation of circulating neutrophils in ALS, consistent with previous reports. For instance, calprotectin subunits S100A8 and S100A9 were increased in participants with ALS in two separate whole blood RNA‐seq datasets [11, 41]. Similarly, MMP9 was increased in participants with ALS in whole blood RNA‐seq [34], and both pro‐ and active forms of MMP9 are elevated in ALS serum [42], as is MMP9 enzymatic activity [42, 43]. Plasma NGAL is also increased in ALS [44, 45, 46]. Previous studies on circulating DNA in ALS are conflicting; however, several studies reported no difference in DNA levels [47, 48], while another reported elevated circulating DNA in ALS [40]. This is likely due to the small sample sizes in these studies, as even with over 400 participants, we only observed a trend toward increased dsDNA. Additionally, a recent study identified that plasma levels of neutrophil markers, including MMP9, were associated with an increased risk of developing ALS, suggesting that changes in neutrophil function likely precede clinical onset [46]. Collectively, these findings suggest that critical aspects of neutrophil function are increased in ALS compared to controls.

Finally, we examined whether neutrophil function markers were associated with ALS survival. In a case‐only analysis, we found that calprotectin, MMP9, and NGAL levels in the plasma did not associate with ALS survival. These findings are consistent with previous studies, which found no link between MMP9 levels [42] or NGAL (also known as lipocalin‐2) levels [45] and ALS duration. In contrast, we found that increased dsDNA levels were associated with reduced ALS survival, but only in female participants, suggesting a role for NET formation in ALS progression. Though little is known about NET formation and its association with ALS, our findings are unsurprising, as we have previously demonstrated that genes associated with NET formation are upregulated in the spinal cords of SOD1^G93A^ ALS mice [35] and are dysregulated in individuals with ALS [36]. Moreover, NETs have been implicated in the pathogenesis of other neurodegenerative diseases. For instance, in Alzheimer's disease, serum NET concentration is elevated relative to controls [23], and NET formation has been observed in the cerebral vasculature [18] and parenchyma [33]. In mouse models, cognition was also improved when neutrophil accumulation and NET formation were blocked in the cerebral vasculature [33]. Our findings therefore suggest that NET formation may play a similar role in ALS pathogenesis, but further studies are needed.

Interestingly, many of the differences observed in the study were sex‐specific. Perhaps most striking, associations between dsDNA levels and reduced ALS survival were driven entirely by female participants. We have observed this female‐specific effect in our previous studies. For instance, we showed that peripheral neutrophil levels are associated with reduced survival in ALS, but this association is only true in female participants [8], and that neutrophil levels were associated with disease progression, but only in older females with ALS [3]. Similarly, a recent study reported a positive association between neutrophil levels and increased lower motor neuron dysfunction in ALS, and this association was stronger in females than in males [49]. In contrast, a recent study associated neutrophil function markers in plasma with an increased risk of ALS, but this association was only observed in males [46]. Sex‐based differences have been reported in other immune populations in ALS as well, including the associations between clinical metrics of ALS and monocyte levels, the platelet: Lymphocyte ratio, and the systemic inflammatory index [17, 50]. Though the underlying mechanisms have not been established, sex differences are well documented in ALS, with females having a higher proportion of bulbar onset, older age at onset, slower decline in weight and respiratory function, and longer survival [1, 51]. One possibility is a relationship with sex hormones. For example, we recently showed that elevated 11‐ketotestosterone levels were associated with an increased risk of ALS, and that estrone and estradiol were associated with shorter survival in females [52]. Interestingly, estradiol modulates NET formation in neutrophils in vitro, but its impact is sex‐dependent: In neutrophils collected from females, NET formation is increased by estradiol, while in neutrophils from males it is suppressed [53, 54]. Together, this indicates that the role of neutrophils and the immune system in ALS likely differs by sex, necessitating further consideration of sex in ongoing research and therapeutic development.

### 4.1 Limitations

The current study has limitations. The ex vivo analysis of NET formation was limited by sample size and the logistics of working with isolated primary neutrophils, as the anticoagulant, isolation method, time, material, and temperature can all affect neutrophil activation [25, 26, 55, 56]; this limitation was minimized, however, by batch corrections and the subsequent neutrophil function marker analyses. With regards to the neutrophil function marker analyses, neutrophils are the primary source of calprotectin, MMP9, NGAL, and dsDNA in the periphery [39, 40], but expression is not limited to neutrophils [39, 40]; however, the correlation analysis between functional markers and neutrophil levels suggests that neutrophils are the source of these markers in the plasma, mitigating this limitation. The phenotype of circulating neutrophils may also not accurately represent the phenotype of tissue‐infiltrating neutrophils, as migration into the tissue seems to increase neutrophil activation and maturation [57]; while direct analysis of CNS tissue is impossible, the limitation is mitigated by the ex vivo analysis of neutrophil NET formation. Both the ex vivo NET analysis and the neutrophil function marker analyses were cross‐sectional rather than longitudinal; future studies will need to examine changes in these markers over the entire course of disease. The plasma analysis was performed on samples that were closer to participant diagnosis than the ex vivo analysis (2.3 m vs. 8.3 m); this is partially mitigated by the similar ALSFRS‐R at the time of analysis (36.0 vs. 34.0) (Table S2). There was a significant age difference between controls and participants with ALS for the neutrophil function marker cohort; this was addressed through comparison of unadjusted, adjusted, and age‐matched logistic regressions, with similar results across all models. Lastly, this is a single‐center study, and no validation cohort or neurodegenerative disease cohort was included. While the large sample sizes reported are a strength, future validation of these findings and inclusion of neurodegenerative disease controls would be valuable. The study has important strengths, however. The ex vivo experiments validate the previous RNA‐Seq findings that NET formation is increased in ALS [11, 35, 36]. Moreover, our neutrophil function marker analysis included a robust cohort of 178 participants with ALS, allowing us to stratify the analyses by sex and conduct robust survival models with multiple key clinical covariates included to minimize confounding effects.

## 5 Conclusion

Neutrophils have long been implicated in ALS, though little is known about the underlying mechanisms contributing to ALS pathology. Here, we demonstrated that neutrophil function is altered in ALS and that NET formation may contribute to disease progression, particularly in females. These findings support continued research into the role of neutrophils and NET formation in ALS and suggest they may serve as potential therapeutic targets.

## Author Contributions

L.A.B. designed the study, collected data, validated data, designed the statistical approaches, and drafted the manuscript. H.M. designed the study, collected data, validated data, and helped draft the manuscript. J.P. and D.J. validated data, designed the statistical approaches, and drafted the manuscript. S.J.T., I.F.W.‐D., and A.D.C. collected data, validated data, and helped draft the manuscript. E.L.F. designed the study, designed the IRB protocol, and drafted the manuscript. S.A.G. designed the study, validated the data, designed the IRB protocol, designed the statistical approaches, and drafted the manuscript. B.J.M. designed the study, validated the data, designed the statistical approaches, and drafted the manuscript. L.A.B., J.P., D.‐G.J., E.L.F., S.A.G., and B.J.M. had unrestricted access to the data. The manuscript was primarily drafted by L.A.B., E.L.F., S.A.G., and B.J.M. with feedback from all authors. All authors agreed to submit the manuscript, read and approved the final draft, and take responsibility for the accuracy of the data and the analyses.

## Funding

This work was supported by CDC/ATSDR (R01TS000339, U01TS000351), the National Institutes of Health (F31NS139629, R01ES030049, R01NS120926, R01NS127188, T32AI007413), the Richard Stravitz Foundation, the Peter R. Clark Fund for ALS Research, the Coleman Therapeutic Discovery Fund, the Stanford Morris ALS Research Fund, the Michael R. Johns Fund, the Robert A. Epstein and Joan M. Chernoff‐Epstein Emerging Scholar Fund, NeuroNetwork for Emerging Therapies, the Scott Pranger ALS Center, the Dr. Randall Whitcomb Fund for ALS Genetics, the Coleman Therapeutic Discovery Fund, James and Margaret Hiller, Eric and Linda Novak.

## Conflicts of Interest

Drs. Murdock, Goutman, and Feldman are listed as inventors on a patent, Issue number US10660895, held by the University of Michigan titled “Methods for Treating Amyotrophic Lateral Sclerosis” that targets immune pathways for use in ALS therapeutics. The other authors report no disclosures relevant to this manuscript.

## Supporting information


**Figure S1:** Neutrophil function marker data curation and validation. (A) Calprotectin, MMP9, NGAL, and dsDNA were measured for 418 plasma samples. Measurements with a coefficient of variation (CV) greater than 20 were excluded; 7 samples had all measurements excluded and were removed from the cohort. Measurements outside the limit of detection (LOD) were imputed as the LOD; outlying measurements, defined as Q3 + 3xIQR, were imputed as Q3 + 3xIQR. (B) Concentrations of calprotectin, MMP9, NGAL, and dsDNA for the internal control across 15 plates. Plasma from 3 separate donors was pooled and aliquoted, with a freshly thawed aliquot included on each plate in duplicate. Duplicates were averaged, and concentration was calculated using the standard curve from that plate. Concentrations for all plates are plotted. The median is indicated with a dotted line, and the IQR is shaded (C, D). The distribution of marker concentrations was compared between all measurements with a CV > 20 excluded (raw), those measurements after normalization using mean‐centering based on the internal control (normalized), and normalized measurements with values outside the LOD and outliers imputed (imputed). Data were stratified by disease status (C) or by processing step (D). *p* values from Kruskal–Wallis test and post hoc Dunn test with Bonferroni correction (C) and Wilcox test (D), * ≤ 0.05, ** ≤ 0.01, *** ≤ 0.001, **** ≤ 0.0001. Abbreviations: CV—coefficient of variation; MMP9—matrix metalloproteinase 9; NGAL—neutrophil gelatinase‐associated lipocalin 2; LOD—limit of detection; dsDNA—double‐stranded DNA; IQR—inter‐quartile range; Q3—quartile 3.
**Figure S2:** Sensitivity analyses for the effect of age and onset segment on ex vivo NET formation. (A) Age sensitivity analysis: Correlations between the proportion of neutrophils forming NETs and age at sample were calculated using Spearman's correlation, stratified by subject type, timepoint, and condition (spontaneous vs. maximal). (B) Onset segment sensitivity analysis: Proportion of neutrophils forming NETs compared between participants with ALS with bulbar, cervical, and lumbar onset. (Respiratory (*n* = 3) and thoracic (*n* = 4) onset segments were excluded due to small sample size). No *p*‐values ≤ 0.05 were identified via a Wilcoxon rank sum test.
**Figure S3:** Correlation of neutrophil function markers with other markers and immune variables. (A) Schema of expected correlations between markers [19–21]. (B) Spearman's rank correlation was used to assess correlations among neutrophil markers and between neutrophil function markers and immune variables. Both controls and participants with ALS were included. Correlation coefficients are included. Only significant correlations are displayed. NLR = neutrophil‐to‐lymphocyte ratio; NMR = neutrophil‐to‐monocyte ratio. *N* = 234.
**Figure S4:** Neutrophil function markers stratified by sex and onset segment. Plasma concentrations of calprotectin, MMP9, NGAL, and dsDNA were stratified by sex and onset segment (bulbar, cervical, and lumbar; respiratory (*n* = 5) and thoracic (*n* = 4) onset segments excluded due to small sample size). No *p*‐values ≤ 0.05 were identified via a Wilcoxon rank sum test.
**Table S1:** Quantification of the proportion of neutrophils forming NETs.
**Table S2:** Comparison of demographics and clinical characteristics of participants with ALS in the NET formation (NET) and plasma neutrophil function marker (plasma) cohorts.
**Table S3:** ALS participant clinical characteristics stratified by sex for plasma neutrophil function marker cohort.
**Table S4:** Quantification of neutrophil function markers in plasma.
**Table S5:** Comparison of demographics and clinical characteristics of controls and participants with ALS in the complete neutrophil function marker (complete) cohort and the age‐matched subsampling (matched).
**Table S6:** Sensitivity analyses for neutrophil function markers to account for age and sex using logistic regressions.

## Data Availability

The data that support the findings of this study are available from the corresponding author upon reasonable request.
