# Supplementary material for: Peripheral Neutrophil Activation and Extracellular Trap Formation in Amyotrophic Lateral Sclerosis
Source: Ann Clin Transl Neurol. 2026 May 14:10.1002/acn3.70431. Online ahead of print. doi: 10.1002/acn3.70431 (PMC13394124; doi:10.1002/acn3.70431)
Supplement: Supplementary file 1 — Figure S1: Neutrophil function marker data curation and validation. (A) Calprotectin, MMP9, NGAL, and dsDNA were measured for 418 plasma samples. Measurements with a coefficient of variation (CV) greater than 20 were excluded; 7 samples had all measurements excluded and were removed from the cohort. Measurements outside the limit of detection (LOD) were imputed as the LOD; outlying measurements, defined as Q3 + 3xIQR, were imputed as Q3 + 3xIQR. (B) Concentrations of calprotectin, MMP9, NGAL, and dsDNA for the internal control across 15 plates. Plasma from 3 separate donors was pooled and aliquoted, with a freshly thawed aliquot included on each plate in duplicate. Duplicates were averaged, and concentration was calculated using the standard curve from that plate. Concentrations for all plates are plotted. The median is indicated with a dotted line, and the IQR is shaded (C, D). The distribution of marker concentrations was compared between all measurements with a CV > 20 excluded (raw), those measurements after normalization using mean‐centering based on the internal control (normalized), and normalized measurements with values outside the LOD and outliers imputed (imputed). Data were stratified by disease status (C) or by processing step (D). p values from Kruskal–Wallis test and post hoc Dunn test with Bonferroni correction (C) and Wilcox test (D), * ≤ 0.05, ** ≤ 0.01, *** ≤ 0.001, **** ≤ 0.0001. Abbreviations: CV—coefficient of variation; MMP9—matrix metalloproteinase 9; NGAL—neutrophil gelatinase‐associated lipocalin 2; LOD—limit of detection; dsDNA—double‐stranded DNA; IQR—inter‐quartile range; Q3—quartile 3. Figure S2: Sensitivity analyses for the effect of age and onset segment on ex vivo NET formation. (A) Age sensitivity analysis: Correlations between the proportion of neutrophils forming NETs and age at sample were calculated using Spearman's correlation, stratified by subject type, timepoint, and condition (spontaneous vs. maximal). (B) Onset segment sen [file ACN3-9999-0-s001.docx]

**Supplemental Tables**

**Supplemental Table 1. Quantification of proportion of neutrophils forming NETs.**

|  |  | **Control** | | **ALS** | |  |
| --- | --- | --- | --- | --- | --- | --- |
| **Time** | **Condition** | **N** | **Median (IQR)** | **N** | **Median (IQR)** | **P-value^1^** |
| **2 hours** | Spontaneous | 19 | 1.0 (0.2-11.9) | 65 | 9.7 (3.0-15.7) | 0.017 |
| **4 hours** | Spontaneous | 20 | 1.4 (0.3-13.3) | 65 | 9.2 (3.3-17.7) | 0.054 |
| **4 hours** | Maximal | 20 | 18.7 (10.7-33.1) | 65 | 15.1 (5.1-23.2) | 0.2 |
| ^1^Wilcoxon rank sum test | | | | | | |

**Supplemental Table 2. Comparison of demographics and clinical characteristics of participants with ALS in the NET formation (NET) and plasma neutrophil function marker (plasma) cohorts.**

|  | **NET**  **(N = 66)** | **Plasma**  **(N = 178)** | **P-value** |
| --- | --- | --- | --- |
| **Sex** |  |  | 0.3^1^ |
| Male | 45 (68%) | 109 (61%) |  |
| Female | 21 (32%) | 69 (39%) |  |
| **Age at sample (years)** | 67.0 (58.9-73.2) | 66.8 (60.5-72.9) | >0.9^2^ |
| **Age at onset (years)** | 64.0 (55.5-70.4) | 65.2 (58.5-71.1) | 0.4^2^ |
| **ALSFRS-R at sample** | 34.0 (27.5-39.5) | 36.0 (30.0-41.0) | 0.14^2^ |
| **Symptom onset to diagnosis (months)** | 11.9 (6.4-24.6) | 11.5 (6.7-18.0) | 0.8^2^ |
| **Diagnosis to sample (months)** | 8.3 (6.5-22.2) | 2.3 (0.8-5.1) | <0.001^2^ |
| **El Escorial Criteria** |  |  | >0.9^1^ |
| Definite | 23 (35%) | 72 (41%) |  |
| Probable | 27 (42%) | 63 (35%) |  |
| Probable, lab supported | 13 (20%) | 37 (21%) |  |
| Possible | 1 (1.5%) | 3 (2%) |  |
| Suspected | 1 (1.5%) | 2 (1%) |  |
| **Onset segment** |  |  | 0.4^1^ |
| Lumbar | 24 (37%) | 71 (40%) |  |
| Cervical | 28 (43%) | 58 (33%) |  |
| Bulbar | 10 (15%) | 41 (23%) |  |
| Thoracic | 1 (1.5%) | 3 (2%) |  |
| Respiratory | 2 (3.5%) | 3 (2%) |  |
| **Race** |  |  | 0.5^1^ |
| White | 62 (94%) | 171 (96%) |  |
| Black or African American | 3 (4.5%) | 5 (3%) |  |
| Asian | 1 (1.5%) | 1 (0.5%) |  |
| American Indian and Alaska Native | 0 (0%) | 1 (0.5%) |  |

N(%); Median (IQR)

^1^Pearson’s Chi-squared test; ^2^Wilcoxon rank sum test

Abbreviations: ALSFRS-R – amyotrophic lateral sclerosis functional rating score – revised; IQR – inter-quartile range

N missing: NET cohort: age at sample – 2, age at onset – 1, ALSFRS-R at sample – 2, Symptom onset to diagnosis – 1, El Escorial criteria – 1, onset segment – 1; neutrophil function marker cohort: age at onset – 3, ALSFRS-R at sample – 1, symptom onset to diagnosis – 6, diagnosis to visit – 4, El Escorial criteria – 1, onset segment - 1

**Supplemental Table 3. ALS participant clinical characteristics stratified by sex for plasma neutrophil function marker cohort.**

|  | **Male (N = 109)** | **Female (N = 69)** | **P-value** |
| --- | --- | --- | --- |
| **Age at sample (years)** | 64.7 (58.3-72.9) | 67.3 (61.8-72.3) | 0.4^1^ |
| **Age at onset (years)** | 63.0 (55.8-70.8) | 66.3 (60.0-71.7) | 0.11^1^ |
| **ALSFRS-R at sample** | 37 (32-41) | 34 (27-40) | 0.035^1^ |
| **Symptom onset to diagnosis (months)** | 12.0 (6.5-18.9) | 11.0 (8.0-16.1) | 0.6^1^ |
| **Diagnosis to sample (months)** | 2.3 (0.8-5.5) | 2.3 (0.8-4.7) | 0.7^1^ |
| **Survival from sample (months)** | 13.7 (6.3-22.6) | 12.4 (8.8-17.2) | 0.5^1^ |
| **Follow-up from sample (months)** | 17.6 (8.6-28.4) | 14.8 (9.3-20.4) | 0.3^1^ |
| **Death event** | 68 (61%) | 44 (64%) |  |
| **El-Escorial Criteria** |  |  | 0.12^2^ |
| Definite | 37 (34%) | 35 (51%) |  |
| Probable | 43 (40%) | 20 (29%) |  |
| Probable, lab supported | 25 (23%) | 12 (17%) |  |
| Possible | 1 (1%) | 2 (3%) |  |
| Suspected | 2 (2%) | 0 (0%) |  |
| **Onset segment** |  |  | 0.018^2^ |
| Lumbar | 42 (39%) | 29 (42%) |  |
| Cervical | 44 (41%) | 14 (20%) |  |
| Bulbar | 19 (17%) | 22 (32%) |  |
| Thoracic | 2 (1.8%) | 2 (2.9%) |  |
| Respiratory | 1 (0.9%) | 2 (2.9%) |  |
| **Race** |  |  | 0.8^2^ |
| White | 105 (96%) | 66 (96%) |  |
| Black or African American | 3 (3%) | 2 (3%) |  |
| American Indian and Alaska Native | 0 (0%) | 1 (1%) |  |
| Asian | 1 (1%) | 0 (0%) |  |

N(%); Median (IQR)

^1^Wilcoxon rank sum test; ^2^Pearson’s Chi-squared test

Abbreviations: ALSFRS-R – amyotrophic lateral sclerosis functional rating score – revised; IQR – inter-quartile range

N missing: Male: 3 age at onset, 1 ALSFRS-R at sample, 4 symptom onset to diagnosis, 3 diagnosis to sample, 1 El Escorial criteria, 1 onset segment; Female: 2 age at onset, 2 symptom onset to diagnosis, 1 diagnosis to sample, 25 survival from sample, 1 family history

**Supplemental Table 4. Quantification of neutrophil function markers in plasma.**

|  |  | **Control** | | **ALS** | |  |
| --- | --- | --- | --- | --- | --- | --- |
|  |  | **N** | **Median (IQR)** | **N** | **Median (IQR)** | **P-value** |
| **Calprotectin** | Overall | 224 | 294 (200-418) | 171 | 372 (272-573) | <0.001**^1^** |
|  | Male | 134 | 298 (196-418) | 104 | 333 (250-495) | 0.111^2^ |
|  | Female | 90 | 288 (206-450) | 67 | 479 (310-668) | <0.001^2^ |
| **MMP9** | Overall | 194 | 106 (78-149) | 142 | 152 (113-237) | <0.001**^1^** |
|  | Male | 119 | 115 (85-163) | 88 | 152 (114-252) | <0.001^2^ |
|  | Female | 75 | 95 (75-131) | 54 | 152 (113-210) | <0.001^2^ |
| **NGAL** | Overall | 218 | 61 (51-74) | 166 | 66 (55-82) | 0.010**^1^** |
|  | Male | 132 | 65 (54-79) | 99 | 69 (56-83) | 0.80^2^ |
|  | Female | 86 | 57 (49-69) | 63 | 65 (52-79) | 0.182^2^ |
| **dsDNA** | Overall | 233 | 0.67 (0.58-0.79) | 178 | 0.69 (0.61-0.80) | 0.064**^1^** |
|  | Male | 140 | 0.69 (0.61-0.80) | 109 | 0.69 (0.63-0.81) | 1.00^2^ |
|  | Female | 93 | 0.64 (0.57-0.74) | 69 | (0.60-0.79) | 0.283^2^ |
| ^1^Wilcoxon rank sum test; ^2^Kruskal-Wallis rank sum test with post-hoc Dunn test using Bonferroni correction | | | | | | |

**Supplemental Table 5. Comparison of demographics and clinical characteristics of controls and participants with ALS in the complete neutrophil function marker (complete) cohort and the age-matched subsampling (matched).**

|  | **Control** | | | | | | **ALS** | | | | | |
| --- | --- | --- | --- | --- | --- | --- | --- | --- | --- | --- | --- | --- |
|  | **Male** | | | **Female** | | | **Male** | | | **Female** | | |
|  | Complete  (N = 140) | Matched  (N = 93) | P | Complete  (N = 93) | Matched  (N = 57) | P | Complete  (N = 109) | Matched  (N = 104) | P | Complete  (N = 69) | Matched  (N = 57) | P |
| **Age at sample (years)** | 62.9  (57.1-70.4) | 65.4  (58.2-72.0) | 0.2^1^ | 60.9  (54.9-67.3) | 65.7  (61.1-69.7) | 0.004^1^ | 64.7  (58.3-72.9) | 64.3  (58.1-72.0) | 0.8^1^ | 67.3  (61.8-72.3) | 66.1  (61.1-71.1) | 0.2^1^ |
| **Race** |  |  | >0.9^2^ |  |  | >0.9^2^ |  |  | >0.9^2^ |  |  | >0.9^2^ |
| White | 136 (97%) | 101 (97%) |  | 91 (98%) | 55 (96%) |  | 105 (96%) | 100 (96%) |  | 66 (96%) | 54 (94%) |  |
| Black or African American |  |  |  |  |  |  | 3 (3%) | 3 (3%) |  | 2 (3%) | 2 (4%) |  |
| Asian | 4 (3%) | 3 (3%) |  | 1 (1%) | 1 (2%) |  | 1 (1%) | 1 (1%) |  |  |  |  |
| American Indian and Alaska Native |  |  |  |  |  |  |  |  |  | 1 (1%) | 1 (2%) |  |
| **Age at onset (years)** |  |  |  |  |  |  | 63.0  (55.8-70.8) | 62.9  (55.5-70.4) | 0.8^1^ | 66.3  (60.0-71.7) | 64.4  (59.5-69.7) | 0.2^1^ |
| **ALSFRS-R at sample** |  |  |  |  |  |  | 37  (32-41) | 37  (32-41) | >0.9^1^ | 34  (27-40) | 33  (25-38) | 0.6^1^ |
| **Symptom onset to diagnosis (months)** |  |  |  |  |  |  | 12.0  (6.5-18.9) | 12.5  (6.5-18.9) | >0.9^1^ | 11.0  (8.0-16.1) | 11.2  (8.3-16.1) | 0.7^1^ |
| **Diagnosis to sample (months)** |  |  |  |  |  |  | 2.3  (0.8-5.5) | 2.4  (0.8-5.5) | >0.9^1^ | 2.3  (0.8-4.7) | 3.0  (0.9-5.0) | 0.5^1^ |
| **El Escorial Criteria** |  |  |  |  |  |  |  |  | >0.9^2^ |  |  | >0.9^2^ |
| Definite |  |  |  |  |  |  | 37 (34%) | 36 (35%) |  | 35 (51%) | 27 (47%) |  |
| Probable |  |  |  |  |  |  | 43 (40%) | 41 (40%) |  | 20 (29%) | 18 (31%) |  |
| Probable, lab supported |  |  |  |  |  |  | 25 (23%) | 24 (23%) |  | 12 (17%) | 10 (18%) |  |
| Possible |  |  |  |  |  |  | 1 (1%) | 1 (1%) |  | 2 (3%) | 2 (4%) |  |
| Suspected |  |  |  |  |  |  | 2 (2%) | 1 (1%) |  |  |  |  |
| **Onset segment** |  |  |  |  |  |  |  |  | >0.9^2^ |  |  | >0.9^2^ |
| Lumbar |  |  |  |  |  |  | 42 (39%) | 40 (39%) |  | 29 (42%) | 27 (47%) |  |
| Cervical |  |  |  |  |  |  | 44 (41%) | 41 (40%) |  | 14 (20%) | 13 (23%) |  |
| Bulbar |  |  |  |  |  |  | 19 (17%) | 19 (18%) |  | 22 (32%) | 14 (24%) |  |
| Thoracic |  |  |  |  |  |  | 2 (2%) | 2 (2%) |  | 2 (3%) | 1 (2%) |  |
| Respiratory |  |  |  |  |  |  | 1 (1%) | 1 (1%) |  | 2 (3%) | 2 (4%) |  |

N(%); Median (IQR)

^1^Wilcoxon rank sum test; ^2^Pearson’s Chi-squared test

Abbreviations: ALSFRS-R – amyotrophic lateral sclerosis functional rating score – revised; IQR – inter-quartile range

N missing: Control female complete: race – 1; Control female matched: race – 1; ALS male complete: age at onset – 1, ALSFRS-R at sample – 1, Symptom onset to diagnosis – 4, diagnosis to sample – 3, El Escorial criteria – 1, onset segment – 1; ALS male matched: age at onset – 1, ALSFRS-R at sample – 1, symptom onset to diagnosis – 4, diagnosis to sample – 3, El Escorial criteria – 1, onset segment – 1; ALS female complete: age at onset – 2, symptom onset to diagnosis – 2, diagnosis to visit – 1; ALS female matched: age at onset – 2, symptom onset to diagnosis – 2, diagnosis to visit – 1

**Supplemental Table 6. Sensitivity analyses for neutrophil function markers to account for age and sex using logistic regressions.**

|  |  | **Unadjusted^1^** | | **Adjusted^1^** | | **Matched^2^** | |
| --- | --- | --- | --- | --- | --- | --- | --- |
| **Marker** | **Group** | **OR (95% CI)** | **P-value^3^** | **OR (95% CI)** | **P-value^3^** | **OR (95% CI)** | **P-value^3^** |
| **Calprotectin** | Overall | 1.63 (1.31, 2.06 | <0.001 | 1.56 (1.27, 1.95) | <0.001 | 1.65 (1.29, 2.17) | <0.001 |
|  | Male | 1.42 (1.05, 1.96) | 0.026 | 1.39 (1.03, 1.91) | 0.035 | 1.35 (0.97, 1.92) | 0.086 |
|  | Female | 1.88 (1.36, 2.68) | <0.001 | 1.80 (1.33, 2.52) | <0.001 | 2.13 (1.44, 3.40) | <0.001 |
| **MMP9** | Overall | 2.10 (1.62, 2.78) | <0.001 | 2.11 (1.63, 2.79) | <0.001 | 2.12 (1.58, 2.94) | <0.001 |
|  | Male | 1.81 (1.35, 2.50) | <0.001 | 1.82 (1.36, 2.51) | <0.001 | 1.72 (1.26, 2.44) | 0.001 |
|  | Female | 3.07 (1.80, 5.87) | <0.001 | 3.19 (1.90, 6.00) | <0.001 | 5.25 (2.40, 14.11) | <0.001 |
| **NGAL** | Overall | 1.27 (1.03, 1.58) | 0.030 | 1.34 (1.09, 1.65) | 0.006 | 1.20 (0.95, 1.52) | 0.134 |
|  | Male | 1.21 (0.93, 1.58) | 0.162 | 1.24 (0.96, 1.61) | 0.101 | 1.11 (0.84, 1.48) | 0.472 |
|  | Female | 1.43 (0.98, 2.13) | 0.069 | 1.56 (1.09, 2.30) | 0.019 | 1.40 (0.93, 2.19) | 0.119 |
| **dsDNA** | Overall | 1.16 (0.95, 1.42) | 0.141 | 1.13 (0.93, 1.37) | 0.227 | 1.21 (0.97, 1.52) | 0.094 |
|  | Male | 1.10 (0.83, 1.46) | 0.513 | 1.08 (0.82, 1.43) | 0.598 | 1.05 (0.77, 1.43) | 0.754 |
|  | Female | 1.23 (0.91, 1.66) | 0.180 | 1.18 (0.89, 1.57) | 0.246 | 1.45 (1.03, 2.14) | 0.043 |
| **^1^**Complete dataset; ^2^age-matched subsample  ^3^Logistic regression | | | | | | | |

**Supplemental Figures**


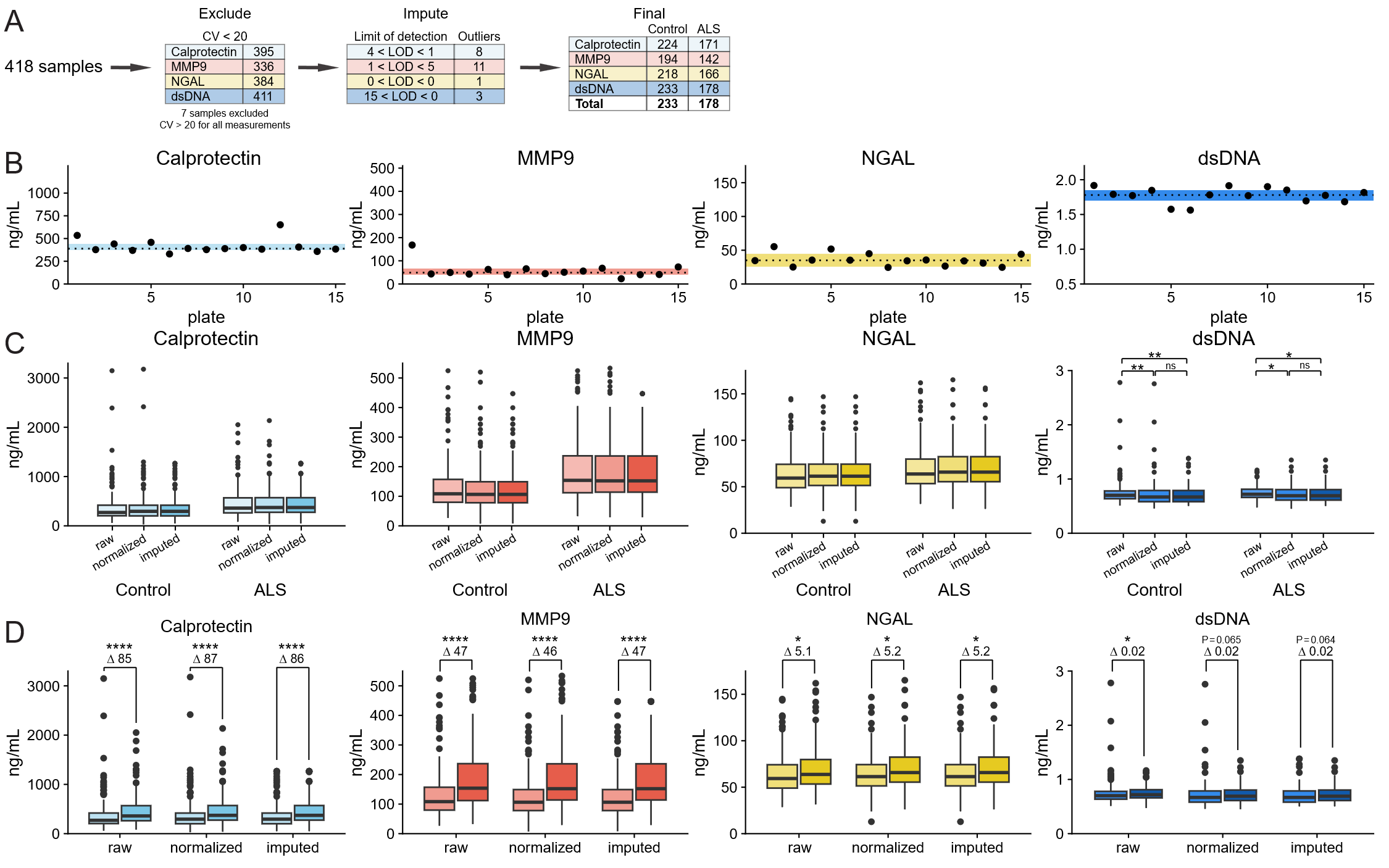


**Supplemental Figure 1. Neutrophil function marker data curation and validation.** (**A**) Calprotectin, MMP9, NGAL, and dsDNA were measured for 418 plasma samples. Measurements with a coefficient of variation (CV) greater than 20 were excluded; 7 samples had all measurements excluded and were removed from the cohort. Measurements outside the limit of detection (LOD) were imputed as the LOD; outlying measurements, defined as Q3 + 3xIQR, were imputed as Q3 + 3xIQR. (**B**) Concentrations of calprotectin, MMP9, NGAL, and dsDNA for the internal control across 15 plates. Plasma from 3 separate donors was pooled and aliquoted, with a freshly thawed aliquot included on each plate in duplicate. Duplicates were averaged and concentration was calculated using the standard curve from that plate. Concentrations for all plates are plotted. The median is indicated with a dotted line, and IQR is shaded. (**C, D**) The distribution of marker concentrations was compared between all measurements with a CV > 20 excluded (raw), those measurements after normalization using mean-centering based on the internal control (normalized), and normalized measurements with values outside the LOD and outliers imputed (imputed). Data were stratified by disease status (**C**) or by processing step (**D**). P values from Kruskal-Wallis test and post-hoc Dunn test with Bonferroni correction (**C**) and Wilcox test(**D**), * ≤ 0.05, ** ≤ 0.01, *** ≤ 0.001, **** ≤ 0.0001. Abbreviations: CV – coefficient of variation; MMP9 – matrix metalloproteinase 9; NGAL – neutrophil gelatinase associated lipocalin 2; LOD – limit of detection; dsDNA – double-stranded DNA; IQR – inter-quartile range; Q3 – quartile 3


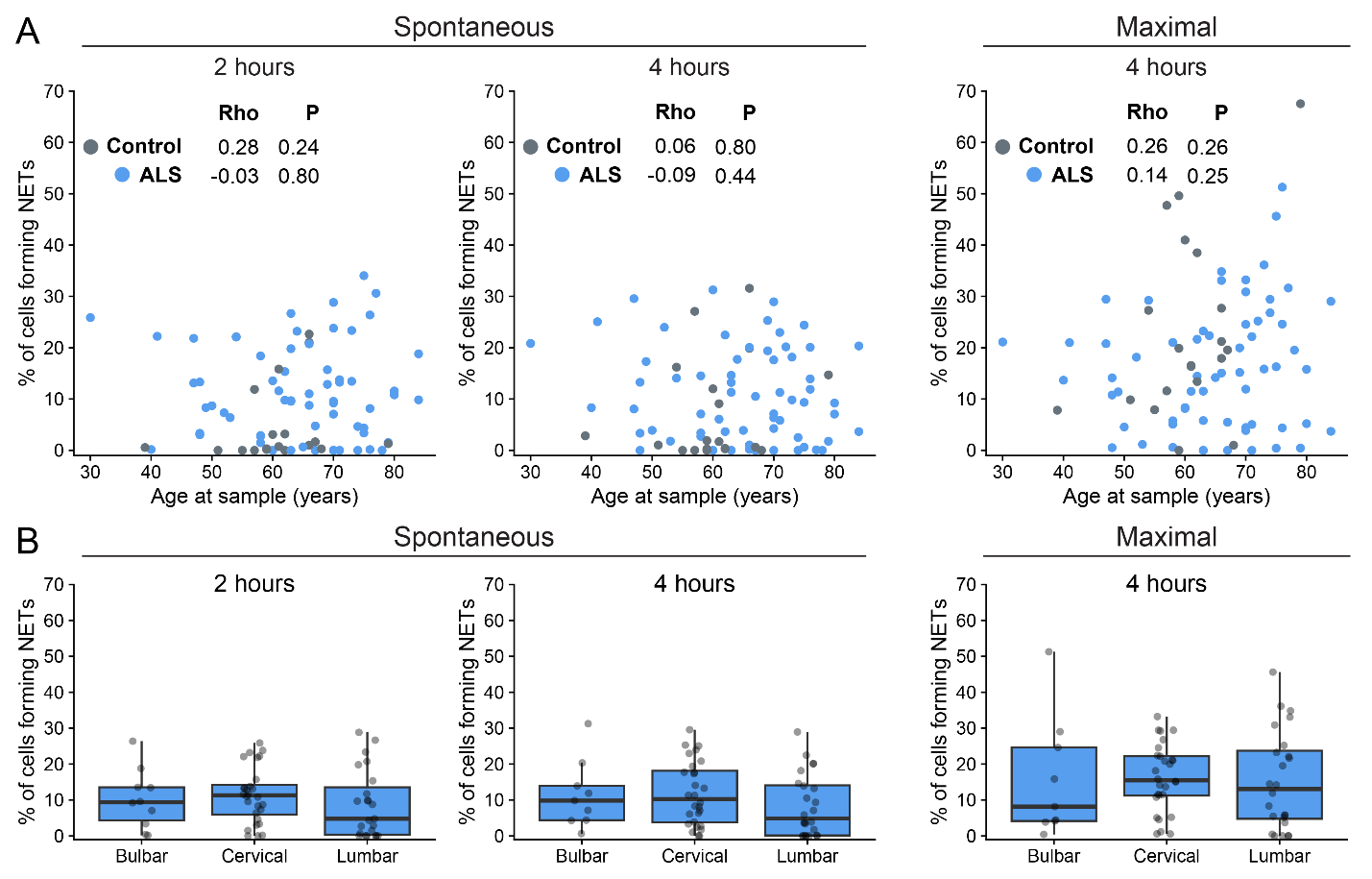


**Supplemental Figure 2. Sensitivity analyses for effect of age and onset segment on *ex vivo* NET formation.** (**A**) Age sensitivity analysis: Correlations between the proportion of neutrophils forming NETs and age at sample were calculated using Spearman’s correlation, stratified by subject type, timepoint, and condition (spontaneous vs maximal). (**B**) Onset segment sensitivity analysis: Proportion of neutrophils forming NETs compared between participants with ALS with bulbar, cervical, and lumbar onset. (Respiratory (n=3) and thoracic (n=4) onset segments excluded due to small sample size). No p values ≤ 0.05 were identified via a Wilcoxon rank sum test.


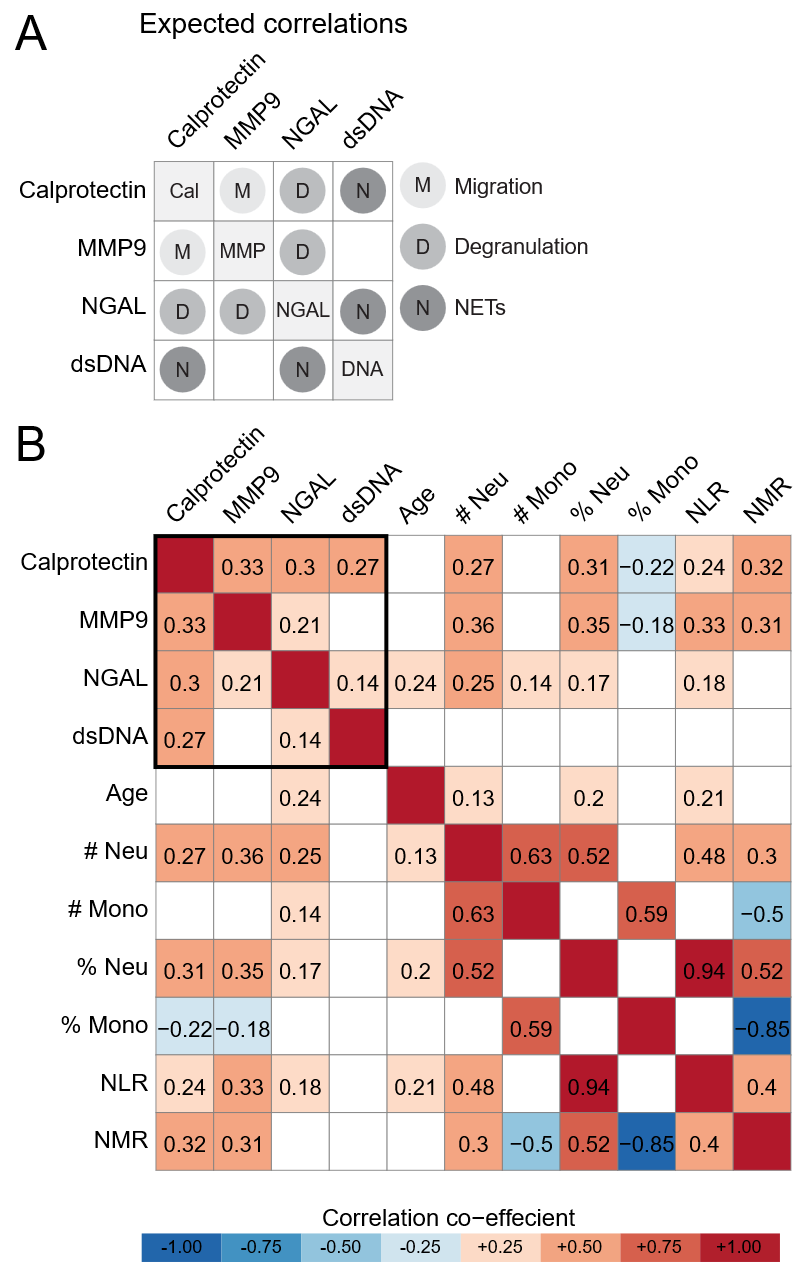


**Supplemental Figure 3. Correlation of neutrophil function markers with other markers and immune variables.** (**A**) Schema of expected correlations between markers^18-20^. (**B**) Spearman rank correlation was used to assess correlations among neutrophil markers and between neutrophil function markers and immune variables. Both controls and participants with ALS were included. Correlation coefficients are included. Only significant correlations are displayed. NLR = neutrophil-to-lymphocyte ratio; NMR = neutrophil-to-monocyte ratio. N = 234.


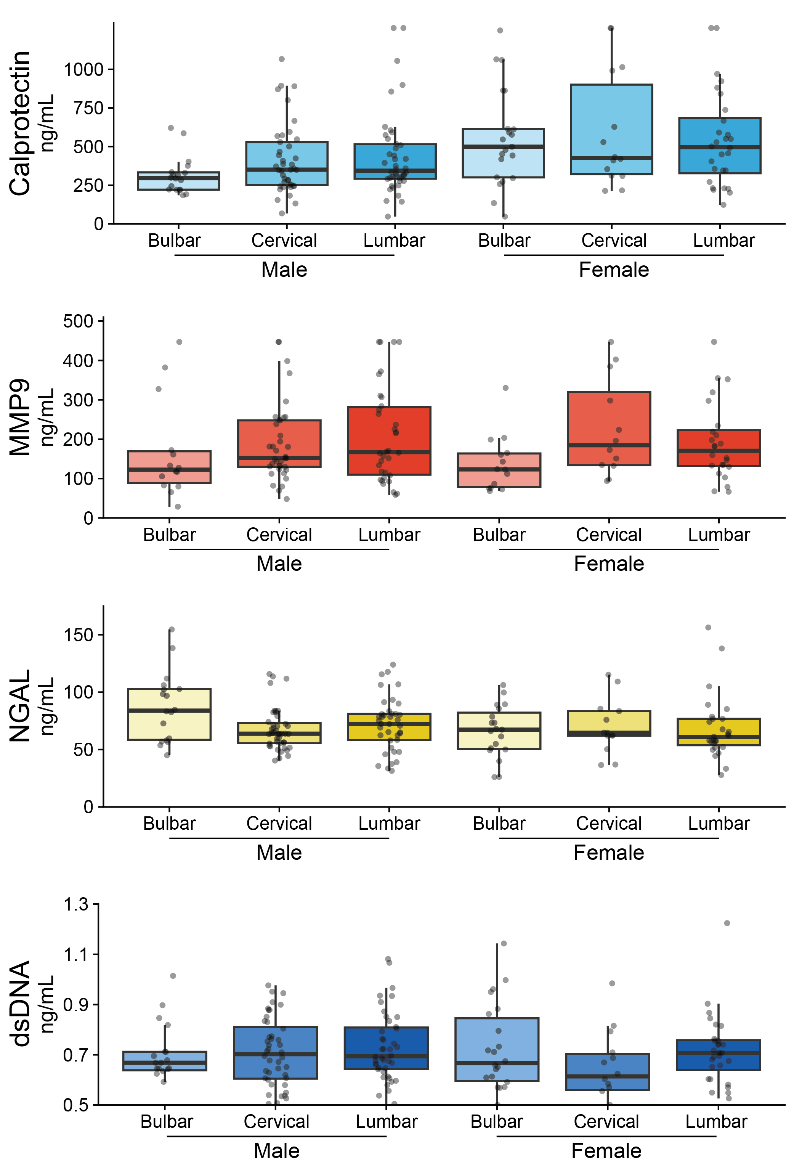


**Supplemental Figure 4. Neutrophil function markers stratified by sex and onset segment.** Plasma concentrations of calprotectin, MMP9, NGAL, and dsDNA were stratified by sex and onset segment (bulbar, cervical, and lumbar; respiratory (n=5) and thoracic (n=4) onset segments excluded due to small sample size). No p values ≤ 0.05 were identified via a Wilcoxon rank sum test.
